# Prevalence of symptoms suggestive of sleep apnea among children and its impact on academic performance

**DOI:** 10.1186/s42506-025-00182-2

**Published:** 2025-02-03

**Authors:** Silvia D. Boyajian, Muna A. Salameh, Kholoud Alzyoud, Enas A. Amaireh, Lujayn Badah, Malek Al Qutami, Mira Alsharayri, Osama Abubaker, Rzan Shwashreh

**Affiliations:** 1 Department of Basic Medical Sciences, Faculty of Medicine, Al-Balqa Applied University, Al-Salt, 19117 Jordan; 2 Department of Medical Imaging, Faculty of Applied Medical Sciences, The Hashemite University, Zarqa, 13133 Jordan

**Keywords:** Pediatric obstructive sleep apnea, Sleep-related breathing disorder, Nocturnal enuresis, School performance

## Abstract

**Background:**

Obstructive sleep apnea (OSA) is a common condition in pediatrics that requires prompt recognition and management to minimize its negative impact on their health and development. Data on the prevalence of OSA among school-age minors in Jordan is scarce. This study aims to estimate the prevalence of OSA in children and adolescents and identify factors that make them more likely to have OSA and its impact on academic achievement.

**Methods:**

This school‐based cross‐sectional study was conducted from January to March 2023. A questionnaire including information on demographic variables, school performance, and a validated sleep-related breathing disorder (SRBD) scale for pediatrics was distributed to 1798 students in schools all over Amman. The prevalence of OSA (defined as SRBD score > 33%) was reported as a proportion along with its 95% confidence interval.

**Results:**

Out of the 2000 questionnaires distributed, 1798 were analyzed. Among these, 1079 (60%) were from children aged 5 to 10 years, and 719 (40%) were from adolescents aged 11 to 18 years. The prevalence of high risk for OSA (HR-OSA) was higher among adolescents than in children (25.6% vs. 20.8%). Adolescents with unemployed fathers were more likely to be HR-OSA (33.70 vs 17.57%, *p* < 0.001). In contrast, paternal employment did not affect the prevalence of HR-OSA in children. Sleep talking, bruxism, and sweating during sleep were more common among children with positive SRBD. Nocturnal enuresis (NE) was associated with positive SRBD in children and adolescents. HR-OSA was associated with poor academic performance in both study groups.

**Conclusion:**

HR-OSA is highly prevalent in Jordanian children and adolescents. HR-OSA can occur alongside other sleep disorders, and it significantly impairs the academic performance of affected individuals. This study found an association between high risk for OSA and other sleeping disorders (sleep talking, sleep bruxism, and sweat during sleeping) and nocturnal enuresis which needs to be confirmed in larger studies.

## Introduction

Obstructive sleep apnea (OSA) is a sleep-related breathing disorder characterized by episodic partial or complete upper airway obstruction, disrupting standard sleep patterns, and altering gas exchange [1]. Globally, an estimated one billion adults have OSA, and 425 million have moderate-to-severe OSA, which requires treatment [2]. OSA can also occur in 2–5% of children [3]. Notwithstanding, the prevalence of OSA appears to be increasing and may be underestimated, given the continuously increasing rates of obesity [4]. The American Academy of Pediatrics recently restated its recommendation that children showing signs and symptoms indicative of OSA should have a polysomnography (PSG) to assess and manage the condition appropriately [5].

In otherwise healthy children, obesity and different anatomical factors, such as decreased dimensions of the oropharynx, are the major risk factors for OSA [6]. The body mass index (BMI) and the likelihood of developing OSA have a well-established relationship [7]. OSA affects more than 40% of people with a BMI greater than 30 and 60% of people with metabolic syndrome [8]. Furthermore, the severity of OSA was found to be directly related to changes in body weight [9]. Anatomical variations, such as the size of the mid and lower face skeleton, upper airway tissue, and tongue, are key factors in childhood OSA [10]. In children, adenotonsillar hypertrophy is a widely recognized risk factor for OSA and plays a key role in its pathogenesis. It is considered the most common cause of upper airway constriction in this age group [6]. The presence of medical, neurological, or skeletal conditions that reduce airway size, impair collapsibility, or neural control of the upper airway may increase the risk of OSA development. These conditions include cerebral palsy, craniofacial abnormalities, Down syndrome, Prader-Willi-syndrome, and neuromuscular disorders [11]. Other risk factors present antenatally or during infancy may also increase the risk of developing OSA later in life [12].

Clinical manifestations of OSA in children can be broadly classified into nocturnal and daytime symptoms. Various nocturnal symptoms have been reported, including snoring, labored breathing, and restless sleep [13]. Some less well-recognized symptoms include nocturnal enuresis (NE), nocturia, and parasomnias such as sleepwalking, sleep terrors, or confusional awakenings [14]. Daytime sleepiness, a common feature of OSA in adults, might manifest in children as frequent daytime napping or falling asleep at school, on the school bus, or during short car rides [6]. Furthermore, children with OSA exhibit higher levels of inattention and behavioral problems such as aggressiveness, impulsivity, and hyperactivity [15]. OSA has devastating consequences if left untreated, and it is associated with several cardiovascular problems that may include systemic hypertension, ventricular dysfunction [16], and metabolic syndrome [11]. The consequences of OSA are not limited to medical problems; children with OSA frequently exhibit learning difficulties and have lower academic grades [17], and pediatric OSA has been linked to lower academic achievement [18]. Poor academic performance is a notable complication of OSA, potentially attributable to cortical and sympathetic arousals and hypoxemia, which can affect memory consolidation [18]. In 2015, a meta-analysis conducted to assess the relationship between sleep-disordered breathing (SDB) and academic performance in school-age children, revealed a significant association with poorer academic outcomes [19]. Another study investigated the prevalence of OSA among 5- to 10-year-old school children and its impact on academic achievement and found that students who tested positive on the sleep-related breathing disorder (SRBD) questionnaire were more likely to have lower grades than those who tested negative [17]. A study assessing the prevalence of OSA in children and adolescents is crucial as it sheds light on the magnitude of the problem, especially given that no such study has previously been published in Jordan. This study will provide vital information on the health burden of OSA in this population and will help guide healthcare policies and decisions to address the issue. Moreover, early detection and intervention of OSA in children and adolescents can significantly impact their overall health, quality of life, and academic and social development. After a thorough review of the literature, this study is, our knowledge, the first in Jordan to highlight the correlation between OSA and academic performance in Jordanian children and adolescents. This study aims to investigate the prevalence of HR-OSA among children and adolescents in Amman, Jordan, and its links to sleep disorders—such as enuresis, socioeconomic factors, and school grades.

## Methods

### Study design and participants

A total of 1798 children participated in this cross-sectional study, conducted from January 2023 to March 2023. The study included Jordanian children aged 5 to 18 years of both genders. Children were categorized into two groups: young children (5–10 years old) and adolescents (11–18 years old). The age groups were chosen based on the literature [17, 20, 21]. Non-Jordanian children and those outside this age range were excluded from the study.

This study was approved by the local ethics committee of Al-Balqa Applied University and the Ministry of Education (number: 707/18/3/2/18), date: 18/01/2023).

### Sample size

The sample size of this study was 1800 individuals. The reported prevalence of OSA using a questionnaire approach is approximately 3**–**10% [17, 20, 22]. Accordingly, the required sample size to obtain a prevalence of 10% with a 20% relative error and a design effect of 2 would be 1552 individuals. Thus, 1800 participants are sufficient for this study.

### Sampling technique

The current study utilized simple random sampling, where the questionnaire was distributed to randomly selected private and governmental schools according to the geographical divisions of Amman: north, south, east, and west.

### Questionnaire

The questionnaire was formatted as an online Google Form. Parents were targeted through lists provided by the school administration and the questionnaire was distributed to the children’s parents along with a participant information form and a consent form through various social media platforms. Participation in the study was voluntary.

The questionnaire gathered information on sociodemographic variables, frequency of NE, and sleeping habits. Additionally, the questionnaire assessed the child's general academic performance and grades in subjects such as Mathematics, Science, Arabic language, English language, Art, and Physical education during the first semester. Academic performance was categorized into the following levels: excellent (scored 90–100), very good (scored 80–89), good (scored 70–79), fair (scored 50–69), and failing (scored less than 50).

Furthermore, the questionnaire included the SRBD scale. The SRBD scale was obtained from the University of Michigan after permission. It consists of 22 symptom items related to snoring frequency, loud snoring, breathing difficulties during sleep, observed apneas, daytime sleepiness, and behaviors such as inattentiveness or hyperactivity. Each item in the scale was correlated with childhood OSA, which was confirmed through polysomnography. The SRBD score produced a sensitivity of 0.85, a specificity of 0.87, and a correct classification for 86% of the subjects [23, 24].

Each item in the SRBD scale had three response options: yes (scored as 1), no (scored as 0), or do not know (considered as missing data). The proportion of items reported as positive (yes) was calculated by dividing the number of positive responses by the total number of 22 items. Items with missing or do not know responses were excluded from the calculation. Therefore, the resulting scale score ranged from 0.0 to 1.0. Scores greater than 0.33 indicated a positive result, suggesting a high risk for pediatric SRBD and serving as an indicator for OSA.

### Translation of sleep-related breathing disorder scale

The questionnaire was initially translated from English to Arabic to ensure linguistic accuracy and cultural relevance. The Arabic translation was conducted with appropriate permission. Four bilingual pediatricians were given the English version of the questionnaire and tasked with translating it into Arabic. The Arabic version was then back-translated into English by another four bilingual pediatricians. Any differences between the two versions were subsequently resolved through consensus. The questionnaire underwent a validation process through a pilot test which was administered to parents of 30 children attending a pediatric outpatient department. They were asked to complete the questionnaire and report any uncertainties regarding the questions. Based on their feedback, a final Arabic version of the scale was developed. The translated questionnaire, consisting of 22 items, demonstrated adequate reliability with a Cronbach’s alpha coefficient of 0.67. Participants who took part in the pilot test were subsequently excluded from the main study.

### Statistical analysis

SPSS version 22 (IBM Inc.) was used for data analysis. Data were presented as numbers and percentages. The comparison between different variables among positive and negative OSA children was done using the Chi-square test. A univariate logistic regression analysis was performed to assess the relationship between variables and OSA. Multivariable analysis results were reported as adjusted odds ratios (ORs) with a 95% confidence interval (CI). A *p*-value of < 0.05 was considered statistically significant.

## Results

### Demographics and characteristics of the participants

Out of the 2000 questionnaires distributed, 1798 were analyzed; the response rate was 89.9%. Among these, 1079 (60%) were from children aged 5 to 10 years, and 719 (40%) were from adolescents aged 11 to 18 (Table 1).

**Table 1:** Distribution of sleep-related breathing disorder score by child’s age, Jordan, 2023 (*n* = 1798)

Variables	Children			Variables	Adolescents		
	SRBD score		*p* value		SRBD score		*p* value
	Positive (> 33%),*n* (%) HR-OSA	Negative (≤ 33%), *n* (%) LR-OSA			Positive (> 33%),*n* (%) HR-OSA	Negative (≤ 33%), *n* (%) LR-OSA	
All	224(20.8)	855(79.2)		All	184(25.6)	535(74.4)	
Child’s age				Adolescent's age			
< 6	12 (5.36)	19 (2.22)	0.001	11–12	5 (2.72)	40 (7.48)	0.009
6–7	25 (11.16)	130 (15.21)		12–13	90 (48.91)	206 (38.50)	
7–8	16 (7.14)	114 (13.33)		14–15	78 (42.39)	232 (43.37)	
8–9	24 (10.71)	85 (9.94)		16–18	11 (5.98)	57 (10.65)	
9–10	147 (65.63)	507 (59.30)					

*SRBD* Sleep-related breathing disorder

Out of all children, 266 (24.65%) were males, and 813 (75.34%) were females, with a male-to-female ratio of 1:3.1. Regarding adolescents, 146 (20.3%) were males, and 573 (79.7%) were females, with a male-to-female ratio of 1:3.9. Most of the children and adolescents were enrolled in governmental schools (61.4% and 66.6% respectively), and the majority of children’s schools were in the Western region (35.7%), while the majority of adolescent’s schools were in the Southern region (47.8%). The results show that over half of the children and adolescents slept 9 h or less (63.7% vs 62.7% respectively). Only 7.9% of children and 10.2% of adolescents had tonsillectomy. Less than half of the parents of children and adolescents held a bachelor’s degree or higher, 40.9% vs 36.2% respectively for the father and 44.2% vs 40.9% respectively for the mother. Although most fathers of children and adolescents had jobs (77.9% vs 78.3% respectively), a lower proportion of mothers of children and adolescents were employed (24.17% vs 25.5% respectively) (Table 2).

**Table 2:** Distribution of sleep-related breathing disorder score by various sociodemographic factors among Jordanian children and adolescents, 2023

Variables	Children (5–10 years old)			Adolescents (11–18 years old)		
	SRBD score		*p* value	SRBD score		*p* value
	Positive (> 33%),*n* (%) HR-OSA	Negative (≤ 33%),*n* (%) LR-OSA		Positive (> 33%),*n* (%) HR-OSA	Negative (≤ 33%),*n* (%) LR-OSA	
All	224 (20.8)	855 (79.2)		184 (25.6)	535 (74.4)	
Gender						
Male	54 (24.11)	212 (24.80)	0.832	25 (13.59)	121 (22.62)	**0.017**
Female	170 (75.89)	643 (75.20)		159 (86.41)	414 (77.38)	
Father education						
Primary education	70 (31.25)	244 (28.54)	0.070	84 (45.65)	155 (28.97)	** ≤ 0.001**
Secondary education	77 (34.38)	246 (28.77)		58 (31.52)	162 (30.28)	
Bachelor’s degree or higher	77 (34.38)	365 (42.69)		42 (22.83)	218 (40.75)	
Father’s employment status						
Employed	164 (73.21)	677 (79.18)	0.059	122 (66.30)	441 (82.43)	** ≤ 0.001**
Unemployed	60 (26.79)	178 (20.82)		62 (33.70)	94 (17.57)	
Mother’s education						
Primary education	60 (26.79)	174 (20.35)	**0.012**	77 (41.85)	130 (24.30)	** ≤ 0.001**
Secondary education	83 (37.05)	285 (33.33)		51 (27.72)	167 (31.21)	
Bachelor’s degree or higher	81 (36.16)	396 (46.32)		56 (30.43)	238 (44.49)	
Mother’s employment status						
Employed	45 (20.09)	215 (25.15)	0.134	40 (21.74)	144 (26.92)	0.154
Unemployed	179 (79.91)	640 (74.85)		144 (78.26)	391 (73.08)	
School type						
Private	76 (33.93)	340 (39.77)	0.085	31 (16.85)	209 (39.07)	** ≤ 0.001**
Governmental	148 (66.07)	515 (60.23)		153 (83.15)	326 (60.93)	
School location						
Eastern region	79 (35.27)	222 (25.97)	0.036	60 (32.61)	76 (14.21)	** ≤ 0.001**
Western region	67 (29.91)	318 (37.19)		26 (14.13)	191 (35.70))	
Northern region	15 (6.69)	68 (7.95)		6 (3.26)	16 (2.99)	
Southern region	63 (28.13)	247 (28.89)		92 (50.0)	252 (47.10)	
Sleep duration						
9 h or below	140 (62.50)	547 (63.98)	0.683	96 (52.17)	355 (66.36)	**0.002**
Above 9 h	84 (37.50)	308 (36.02)		88 (47.83)	180 (33.64)	
Sleep time						
Before 9	45 (20.09)	227 (26.55)	**0.034**	33 (17.93)	63 (11.78)	**0.041**
After 9	179 (79.91)	628 (73.45)		151 (82.07)	472 (88.22)	
Tonsillectomy						
Yes	18 (8.04)	68 (7.95)	0.968	21 (11.41)	53 (9.91)	0.939
No	206 (91.96)	787 (92.05)		163 (88.59)	482 (90.09)	

*SRBD* Sleep-related breathing disorder

### Prevalence of high risk for obstructive sleep apnea and its association with various sociodemographic factors

The study’s findings reveal a significant prevalence of high risk for OSA (HR-OSA), particularly in adolescents, with a higher occurrence in females. The prevalence of HR-OSA was higher in adolescents than in children (25.6%, 95% CI 22–28% vs. 20.8%, 95% CI 18–23%), with a significant association found between age and OSA in both children and adolescents (*p* = 0.001, *p* = 0.009). Adolescent females were more likely to have HR-OSA compared to their male counterparts (86.41% vs. 13.59%, *p* = 0.017). HR-OSA was more prevalent in females than males in children; nevertheless, no significant association between gender and HR-OSA was found in this group (*p* = 0.832) (Table 2).

The study’s findings also highlight the potential impact of parental education and employment on the prevalence of HR-OSA in adolescents. In adolescents, there was a significant association between the father or mother's education level and HR-OSA, with the prevalence of HR-OSA being higher in adolescents whose fathers only received primary education (45.65%, *p* = 0.001), as well as their mother (41.85%, *p* = 0.001). There was however no statistically significant relationship between HR-OSA and the father’s education in children (*p* = 0.07). Parental employment had no significant association with HR-OSA in children; however, adolescents who have unemployed fathers were more likely to have HR-OSA (33.70% vs 17.57%, *p* = 0.001) (Table 2).

### Association of high risk for obstructive sleep apnea with sleep duration and sleep time

Adolescents who slept more than 9 h were more likely to have HR-OSA (47.8% vs 33.64%, *p* = 0.002); however, this difference was not significant in children (*p* = 0.683) (Table 2). Regarding the effect of sleep time on the likelihood of having HR-OSA, adolescents who slept before 9 were more likely to have HR-OSA (17.9% vs. 11.78%, *p* = 0.041). However, children who slept after 9 were more likely to have HR-OSA (79.91% vs 73.45%, *p* = 0.034) (Table 2).

### Association of other sleep disorders with sleep-related breathing disorder

Adolescents with OSA were more likely to exhibit sleep talking (27.72% vs. 13.64%; *p* ≤ 0.001). Furthermore, this group had a higher chance of also having bruxism (20.11% vs. 6.73%; *p* < 0.0001, and sweating during sleeping (51.63% vs. 17.57%, *p* ≤ 0.001). Children likewise showed the same results as adolescents. Sleepwalking, however, was also more likely to be observed in children with HR-OSA (9.37% vs. 2.22%, *p* ≤ 0.001). In children and adolescents with HR-OSA, NE was observed in 66.1% and 69.57% of the cases respectively. This was significantly higher than those with low risk for OSA (LR-OSA) in both study groups (*p* ≤ 0.001) (Table 3).

**Table 3:** Association of other sleep disorders with sleep-related breathing disorders among Jordanian children and adolescents, 2023

Associated sleep abnormalities	Children (5–10 years old)			Adolescents (11–18 years old)		
	SRBD score		*p* value	SRBD score		*p* value
	Positive (> 33%),*n* (%)	Negative (≤ 33%),*n* (%)		Positive (> 33%),*n* (%)	Negative (≤ 33%),*n* (%)	
Sleep talking						
Yes	83 (37.05)	137 (16.02)	** ≤ 0.001**	51 (27.72)	73 (13.64)	** ≤ 0.001**
No	141 (62.95)	718 (83.98)		133 (72.28)	462 (86.36)	
Sleep bruxism						
Yes	51 (22.77)	87 (10.18)	** ≤ 0.001**	37 (20.11)	36 (6.73)	** ≤ 0.001**
No	173 (77.23)	768 (89.82)		147 (79.89)	499 (93.27)	
Sleepwalking						
Yes	21 (9.37)	19 (2.22)	** ≤ 0.001**	11 (5.98)	19 (3.55)	0.302
No	203 (90.63)	836 (97.78)		173 (94.02)	516 (96.45)	
Sweat during sleeping						
Yes	85 (37.95)	134 (15.67)	** ≤ 0.001**	95 (51.63)	94 (17.57)	** ≤ 0.001**
No	139 (62.05)	721 (84.33)		89 (48.37)	441 (82.43)	
Nocturnal enuresis						
Yes	148 (66.07)	508 (59.42)	** ≤ 0.001**	128 (69.57)	230 (42.99)	** ≤ 0.001**
No	76 (33.93)	347 (40.58)		56 (30.43)	305 (57.01)	

*SRBD* Sleep-related breathing disorder

### Association of high risk for obstructive sleep apnea with academic performance

Academic performance was significantly and consistently poor in children and adolescents with HR-OSA compared to those with LR-OSA (*p* ≤ 0.001). Moreover, this was evident in almost all subjects taught in school (Table 4).

**Table 4:** Association of high risk for obstructive sleep apnea with school grades among Jordanian children and adolescents, 2023

Variables	Children (5–10 years old)			Adolescents (11–18 years old)		
	SRBD score		*p* value	SRBD score		*p* value
	Positive (> 33%), *n* (%)	Negative (≤ 33%), *n* (%)		Positive (> 33%), *n* (%)	Negative (≤ 33%), *n* (%)	
Overall school grade						
Excellent	94 (41.97)	522 (61.05)	** ≤ 0.001**	35 (19.02)	218 (40.75)	** ≤ 0.001**
Very good	47 (20.98)	176 (20.59)		31 (16.85)	146 (27.29)	
Good	42 (18.75)	91 (10.64)		53 (28.80)	111 (20.75)	
Fair and lower	41 (18.30)	66 (7.72)		65 (35.33)	60 (11.21)	
Mathematics						
Excellent	86 (38.39)	482 (56.37)	**0.001**	32 (17.39)	190 (35.51)	** ≤ 0.001**
Very good	47 (20.98)	185 (21.64)		31 (16.85)	142 (26.54)	
Good	35 (15.63)	103 (12.05)		55 (29.89)	121 (22.62)	
Fair and lower	56 (25.00)	85 (9.94)		66 (35.87)	82 (15.33)	
Science						
Excellent	86 (38.39)	504 (58.95)	** ≤ 0.001**	36 (19.57)	214 (40.00)	** ≤ 0.001**
Very good	48 (21.43)	195 (22.81)		38 (20.65)	156 (29.16)	
Good	46 (20.54)	91 (10.64)		52 (28.26)	115 (21.50)	
Fair and lower	44 (19.64)	65 (7.60)		58 (31.52)	50 (9.34)	
Arabic language						
Excellent	93 (41.52)	514 (60.11)	** ≤ 0.001**	46 (25.00)	235 (43.93)	** ≤ 0.001**
Very good	53 (23.66)	192 (22.46)		47 (25.54)	159 (29.72)	
Good	37 (16.52)	89 (10.41)		44 (23.92)	98 (18.31)	
Fair and lower	41 (18.30)	60 (7.02)		47 (25.54)	43 (8.04)	
English language						
Excellent	83 (37.06)	471 (55.09)	** ≤ 0.001**	33 (17.93)	228 (42.62)	** ≤ 0.001**
Very good	47 (20.98)	171 (20.00)		40 (21.74)	122 (22.80)	
Good	41 (18.30)	115 (13.45)		42 (22.83)	105 (19.63)	
Fair and lower	53 (23.66)	98 (11.46)		69 (37.50)	80 (14.95)	
Art						
Excellent	152 (67.86)	707 (82.69)	** ≤ 0.001**	110 (59.78)	420 (78.51)	** ≤ 0.001**
Very good	45 (20.09)	99 (11.58)		42 (22.83)	77 (14.39)	
Good	14 (6.25)	29 (3.39)		17 (9.24)	19 (3.55)	
Fair and lower	13 (5.80)	20 (2.34)		15 (8.15)	19 (3.55)	
Physical education						
Excellent	158 (70.54)	709 (82.92)	** ≤ 0.001**	108 (58.70)	410 (76.63)	** ≤ 0.001**
Very good	37 (16.52)	100 (11.70)		40 (21.74)	85 (15.89)	
Good	15 (6.69)	29 (3.39)		21 (11.41)	24 (4.49)	
Fair and lower	14 (6.25)	17 (1.99)		15 (8.15)	16 (2.99)	

*SRBD* Sleep-related breathing disorder

### Logistic analysis of factors associated with obstructive sleeping apnea

Multivariate analysis (Table 5) showed that no sociodemographic factors were identified as risk factors for sleep‑related breathing disorders in children. At the same time, other sleeping disorders were identified as risk factors for sleep‑related breathing disorders, including sleep talking (OR = 2.096, 95% CI 1.425–3.082, *p* < 0.001), sleep bruxism (OR = 1.838, 95% CI 1.187–2.846, *p* = 0.006), sleepwalking (OR = 3.134, 95% CI 1.531–6.449, *p* = 0.002), sweat during sleeping (OR = 2.604, 95% CI 1.812–3.742, *p* < 0.001), and nocturnal enuresis (OR = 6.781, 95% CI 4.231–10.868, *p* < 0.001).

**Table 5:** Logistic regression analysis; dependent variable–sleep‑related breathing disorder > 33.0% among young children

Variables	Univariate (unadjusted)		Multivariate (adjusted)	
	OR (95% CI)	*p*	OR (95% CI)	*p*
Age	1.077 (0.954–1.217)	0.231	1.033 (0.911–1.172)	0.614
Father education	1.173 (0.983–1.400)	0.077	0.989 (0.795–1.230)	0.919
Father’s employment status	1.385 (0.987–1.944)	0.059	1.266 (0.886–1.808)	0.196
Mother’s education	0.759 (0.630–0.915)	**0.004**	0.794 (0.629–1.001)	0.051
Mother’s employment status	1.318 (0.918–1.892)	0.135	1.116 (0.760–1.640)	0.575
Sleep time	1.479 (1.029–2.126)	**0.035**	1.444 (0.969–2.153)	0.071
Sleep talking	3.085 (2.225–4.278)	** < 0.001**	2.096 (1.425–3.082)	** < 0.001**
Sleep bruxism	2.67 (1.823–3.908)	** < 0.001**	1.838 (1.187–2.846)	**0.006**
Sleepwalking	4.552 (2.402–8.626)	** < 0.001**	3.143 (1.531–6.449)	**0.002**
Sweat during sleeping	3.290 (2.373–4.562)	** < 0.001**	2.604 (1.812–3.742)	** < 0.001**
Nocturnal enuresis	6.509 (4.186–10.120)	** < 0.001**	6.781 (4.231–10.868)	** < 0.001**

*OR* odds ratio, *CI* confidence interval.

The only significant sociodemographic risk factor for sleep‑related breathing disorders in adolescents was the father’s education (OR 1.831, 95% CI 0.973–1.617, *p* = 0.004). On the other hand, many other sleeping disorders, including sleep talking (OR 2.019, 95% CI 1.262–3.231, *p* = 0.003), sleep bruxism (OR 2.425, 95% CI 1.385–4.247, *p* = 0.002), sweating while sleeping (OR 4.332, 95% CI 2.949–6.362, *p* < 0.001), and nocturnal enuresis (OR 6.972, 95% CI 2.934–16.565, *p* < 0.001), were considered risk factors, as shown in the multivariate analysis (Table 6).

**Table 6:** Logistic regression analysis; dependent variable–sleep‑related breathing disorder > 33.0% among adolescents

Variables	Univariate (unadjusted)		Multivariate (adjusted)	
	OR (95% CI)	*p*	OR (95% CI)	*p*
Age	0.916 (0.732–1.146)	0.441	1.254 (0.691–1.114)	0.081
Father education	1.599 (1.300–1.968)	**0.000**	1.831 (0.973–1.617)	**0.004**
Father’s employment status	2.415 (1.652–3.531)	**0.000**	0.832 (1.206–2.780)	0.094
Mother’s education	0.686 (0.575–0.819)	**0.000**	1.047(0.671–1.032)	0.840
Mother’s employment status	1.357)0.901–2.044(	0.145	0.877 (0.672–1.632)	0.281
Sleep time	0.622 (0.394–983)	**0.042**	0.665 (0.394–1.122)	0.127
Sleep talking	2.539 (1.694–3.807)	** < 0.001**	2.019 (1.262–3.231)	**0.003**
Sleep bruxism	3.445 (2.107–5.632)	**0.000**	2.425 (1.385–4.247)	**0.002**
Sleepwalking	1.503 (0.690–3.273)	0.305	0.832 (0.333–2.081)	0.695
Sweat during sleeping	4.947 (3.435–7.126)	** < 0.001**	4.332 (2.949–6.362)	** < 0.001**
Nocturnal enuresis	8.519 (3.863–18.783)	** < 0.001**	6.972 (2.934–16.565)	** < 0.001**

* OR* odds ratio, *CI* confidence interval.

## Discussion

The prevalence of HR-OSA among Jordanian children and adolescents was found to be 21% and 25%, respectively. This is more prevalent than what studies from other countries have found. A study involving 340 children aged 6–12 years in Saudi Arabia found a prevalence of 9% of OSA [20], which is comparable to the reported prevalence (9.6%) in an Indian study of 3146 children [17]. Another study on the same cohort in Saudi Arabia found an even lower prevalence of OSA: 3.4% [22]. Three studies with a large sample size of a similar cohort reported OSA prevalence that ranges from 1.5 to 5.7% [25, 26]. The varying prevalence in different studies may be attributed to the difference in the methods used to measure breathing during sleep, the definition used to diagnose, and the different characteristics of the studied population. Notwithstanding, the overwhelming body of evidence suggests that OSA has a prevalence of between 1 and 5%, making it a condition that most primary care practitioners will commonly face [3].

In both study groups, the prevalence of HR-OSA was higher in females than in males. However, no significant association was found between gender and the likelihood of having HR-OSA. Other studies have also investigated the prevalence of OSA in males and females, with no significant association between gender and OSA risk identified, which is consistent with our findings [27, 28]. In contrast, results from other studies concluded that males were more likely to have OSA than females [29, 30].

Our research on sociodemographic factors in both children and adolescents revealed some intriguing findings. While in adolescents the employment of the father and the education level of either parent showed a statistically significant difference, the father’s employment and the mother’s education level did not appear to affect the risk of developing OSA, with only the father’s education being identified as a significant risk factor for OSA. In children, however, the education level of the mother showed a significant association with the risk of OSA. This novel finding adds a new dimension to the understanding of OSA risk factors. In line with our findings, an earlier study reported that maternal education showed the most notable and consistent association with the frequency and severity of OSA in children aged 4 to 17 years. Specifically, children whose mothers had less than a high school education had a much higher risk of developing OSA [31]. In contrast, a study in India found that children of working mothers were more likely to develop OSA, but found no significant difference in terms of the either parent’s education level or the father’s employment status [17].

Our research, in line with previous studies, has shown that socioeconomic status may affect OSA risk in children. This finding is significant as it identifies socioeconomic status as a significant determinant of health disparities, due to its role in influencing exposure to unhealthy living conditions and shaping susceptibility to adverse factors. This underscores the need for further research and interventions to address these health disparities and improve the health outcomes of children and adolescents [32].

Parasomnias are unpleasant or disruptive behavioral or emotional events that mainly occur during sleep [33]. Four types of parasomnias commonly occur in children: sleepwalking, sleep talking, sleep terrors, and enuresis [33]. Apart from sleep talking, the prevalence of other types of parasomnias is low in adolescents [33]. Symptoms of parasomnia are relatively common in those suffering from OSA [34]. In this study, children and adolescents with positive SRBD were more likely to experience sleep talking, which can be attributed to the frequent arousal reactions associated with OSA. This is consistent with findings from earlier research involving the same cohort [17, 35]. Sleepwalking was a common manifestation in children with OSA. Other studies have found that children with positive SRBD are more likely to sleepwalk, which can be attributed to insufficient sleep caused by frequent apneic episodes of OSA and the associated respiratory events [20, 35]. Bruxism can be provoked by stress and has a significant correlation with OSA. In both study groups, tooth-grinding was more prevalent among those with positive SRBD, consistent with prior research findings [17, 20, 35].

Oxidative stress caused by intermittent hypoxia in obstructive sleep apnea alters bladder ultrastructure and function, which can result in bladder instability and detrusor overactivity. In response to these changes, sleep apnea-related nocturia, or voiding symptoms may occur [36]. Nocturnal enuresis is a common manifestation of OSA. Several studies reported a significant correlation between OSA and enuresis [37, 38]. In the current study, the prevalence of NE among participants with positive SRBD was 66.1% and 69.57% for children and adolescents respectively, which is consistent with the reported high prevalence of NE found in children with OSA in earlier studies [17, 20]. Moreover, it highlights the significance of considering OSA and other sleep-disordered breathing disorders as differential diagnoses when evaluating children or adolescents with nocturia.

OSA in children and adolescents has a well-documented negative impact on their development and learning abilities [18]. This study showed a significant impact of HR-OSA on the academic performance of children and adolescents, which was evident in different school subjects. This is consistent with the findings of previous studies, where OSA was significantly associated with poor academic performance in the affected individuals [19, 39].

### Study limitations

A strong point of this study is that it included two different age groups and evaluated the effect of various factors that may contribute to the development of OSA. The results of this study may have a significant impact on awareness, especially at the level of healthcare providers, that OSA has a high prevalence in these age groups, making it a common condition that should be considered in clinical practices. Despite that, this study has several limitations. The study’s cross-sectional design cannot determine the temporal relationship between variables. In addition, using polysomnography to diagnose OSA was not feasible. Therefore, the SRBD questionnaire was used instead, which has proven reliable and valid in identifying children with HR-OSA. Moreover, we could not evaluate the effect of BMI on the likelihood of developing OSA, given the fact that a lot of anthropometric data regarding height was missing.

## Conclusion

OSA risk has a high prevalence among Jordanian children and adolescents, 21% and 25%, respectively. It is associated with other sleep disorders such as sleepwalking, sleep talking, bruxism, and nocturnal enuresis. In addition, HR-OSA is associated with poor academic performance in all school subjects. This study found an association between high risk for OSA and other sleeping disorders (sleep talking, sleep bruxism, and sweat during sleeping) and nocturnal enuresis which needs to be confirmed in larger studies.

## Data Availability

The data underlying this article will be shared at a reasonable request by the corresponding author.
